# Epidemiology of Acute Poisonings in Northwestern Syria: A One-Year Study

**DOI:** 10.1055/s-0045-1809706

**Published:** 2025-06-17

**Authors:** Wasim Zakaria, Ahmad Hmaideh, Ahmad Musa Alaaraj, Nour Alsabah Aldan, Mohammad Alabdullah, Mohammed Raafat Alali, Abdalhameed Abdallah, Qusai Rashwani

**Affiliations:** 1 Department of Internal Medicine, Syrian Board of Medical Specialties, Idlib, Syria

**Keywords:** acute poisoning, toxic substances, northwestern Syria, public health, intentional poisoning, conflict zones

## Abstract

**Background:**

Acute poisoning is a significant public health concern, leading to numerous emergency admissions globally. In northwestern Syria, understanding poisoning epidemiology is essential for targeted prevention efforts. This study examines the causes and demographic characteristics of poisoning cases in the region.

**Methods:**

The study was conducted over 1 year (July 1, 2022–July 1, 2023) at six northwestern Syrian hospitals supported by humanitarian organizations. The study gathered information from patients aged 14 years and above who suffered from poisoning during the study period.

**Results:**

Poisoning cases totaled 172 throughout the study period. Majority of patients were females aged between 14 and 24 (76.7 and 55.2%, respectively), most of them were married (65.1%). Most of the patients identified as housewives among the total patient population (52.9%) and had primary or middle school education. A large majority of the population (77.3%) were smokers while most poisoning incidents (77.3%) were reported in rural camps and villages. The study found oral ingestion as the most common route of poisoning at 88.4% and intentional poisonings made up 86% of all cases. The most prevalent toxic agents causing poisoning cases were drugs, 61.6%, with organophosphorus compounds ranking second, 14.5%. The symptom of vomiting appeared most often during acute poisoning cases (48.8%). The administration of specific antidotes took place in 11.6% of patients who needed hospital admission for 94.8% of these cases. The patients stayed in the hospital for an average duration of 33.1 hours. A total of 76.7% of patients achieved full recovery and 7.6% succumbed to their injuries.

**Conclusion:**

Acute poisoning presents as a major health problem across northwestern Syria mostly affecting young married females who live in rural regions. The unusually high number of cases of purposeful poisoning emphasizes the requirement for both psychiatric support and educational programs for the public.

## Introduction

Acute toxic exposure represents a major public health issue, which stands as one of the main factors leading patients to visit emergency units throughout the world. 1 The wide global availability of chemical substances in medicine and industry combined with agricultural usage and everyday life activities results in higher poisoning risks across the world. 2 Acute poisoning develops when a person encounters toxic exposure within 24 hours or less of time. 3 Dangerous poison exposure from accidental and intentional sources results in substantial death rates combined with serious illness across the globe. Different factors determine how poisoning patterns emerge across regions including poison accessibility and how the poison can be obtained as well as the population's economic status and cultural background and religious preferences. Suicidal attempts usually result in increased mortality rates for patients. 4 5 The World Health Organization ranks suicide death as one of the main causes that kills people aged 5 to 29 years old. 4 This study examined poisoning data to describe the demographic features and poisoning causes affecting patients from northwestern Syria by analyzing their admissions to hospital emergency centers. There was an urgent need to study suicide rates in northwestern Syria because of this region's widespread poverty together with ongoing conflict situation. This investigation seeks to generate key knowledge about poisoning patterns in areas facing significant challenges through its assessment of poisoning incidents in northwestern Syria. Acute poisoning research data will direct health professionals toward developing specific management strategies for minimizing the impact of poisoning on this at-risk group.

## Methods

### Study Setting

The research was conducted in humanitarian-supported hospitals in northwestern Syria, specifically: Al-Quds Hospital, Al-Rahma Hospital, Al-Ziraa Hospital, Bab Al-Hawa Hospital, Armanaz Hospital, and Wasim Maaz Hospital. These hospitals also serve as teaching centers under the supervision of the Syrian Board of Medical Specialties, which has been active in the region for several years.

### Study Duration

The research duration extended 12 months from July 1, 2022 to July 1, 2023.

### Sample Selection and Criteria

The study included all patients aged 14 years or older of both sexes who presented to the participating hospitals with acute poisoning during the study period. Individuals who had attended hospitals in the region due to poisoning problems comprised the study participants. The following variables were considered for data collection: age, gender, place of residence, marital status, level of education, type of toxic substance, method of toxic substance exposure, clinical symptoms and vital signs, patient's condition development (including specific treatment requirements), the need for hospital admission, location of admission (standard hospital ward or intensive care unit), duration of hospitalization (if admitted), motivation for ingesting the toxic substance, and the final outcome (recovery, death, or patient's departure from the hospital).

Participants who could not prove their poisoning case through clear evidence, did not know the cause of poisoning, or did not offer essential information about their ingestion motives or substance type or amount were grouped into the “unknown” category.

### Data Collection

The research methods maintained both privacy and autonomy standards for patients. The patients together with their family members provided necessary information whenever patients were conscious or responsive. All collected data remained unidentified to guarantee objectivity in the process. The researchers sought permission from health care authorities before data collection while obtaining consent from all patients through their lawful representatives or patients themselves. An explanation about the research goals and an assurance regarding privacy protection was given to patients. All patients involved in the study accepted the terms to participate. All participants provided their written informed consent for the study according to the established protocol. We received written permission from parents or legal guardians using appropriate ethical guidelines when studying children. For unconscious adults, consent was obtained retrospectively once the patient regained consciousness. In cases where the patient remained unconscious and no legal guardian was available, ethical approval was secured in advance from the responsible health care authority to allow inclusion under emergency research protocols.

### Data Analysis

Prior to analysis the researchers verified that all data was accurate and complete. The first step involved cleaning up and organizing the data set for elimination of inconsistent data as well as missing values. The researchers imported validated data into the Statistical Package for the Social Sciences (SPSS) software for extensive analysis. The researchers computed descriptive measures to present population characteristics data. Standard deviation alongside mean and median values served as statistical measures for continuous variables. Frequency distributions along with percentages demonstrated the data for categorical variables.

## Results

### Demographic Characteristics

**Fig. 1:**
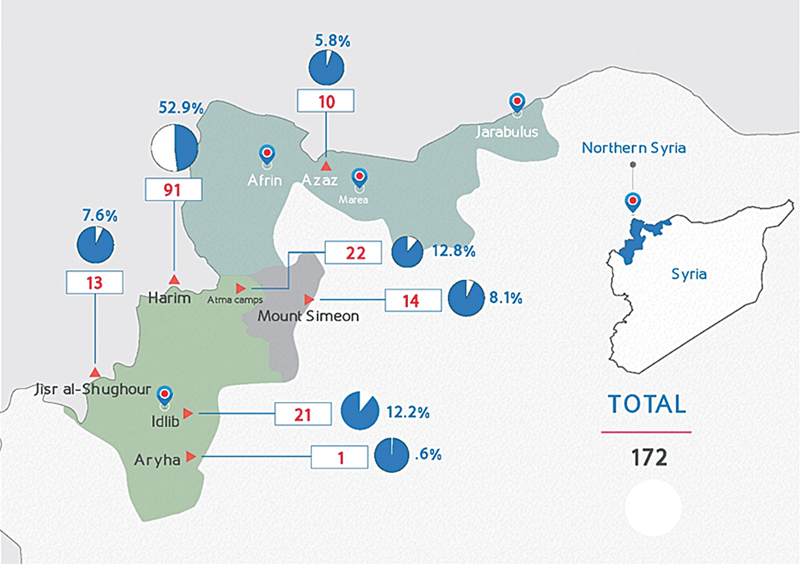
Prevalence of poisoning patient in seven districts in northwest of Syria.

A total of 172 patients visited the emergency departments of northwestern Syria hospitals between July 1, 2022, and July 1, 2023, due to drug overdoses or poisoning events ( Table 1 ). Most affected individuals ( *n* = 95, 55.2%) fell within the 14- to 24-year age category. Out of all patients the female demographic stood as the major population with 132 individuals representing 76.7% of the total. A large proportion of respondents were married, and housewives combined for 65.1 and 52.9%, respectively, of the total survey group. Social support availability and the absence of social networks demonstrate measurable effects on how poisoning incidents occur and how they affect patients. Most participants had completed primary ( *n* = 62, 14.5%) or middle school education ( *n* = 65, 37.8%). Additionally, a considerable percentage of patients were smokers ( *n* = 133, 77.3%). The highest incidence of poisoning was noted in rural areas, particularly among individuals living in camps ( *n* = 133, 77.3%). Fig. 1 shows the prevalence of poisoning patient in seven districts in northwest of Syria. The peak period for poisoning incidents was during the summer ( *n* = 66, 38.4%). The primary method of poisoning involved oral consumption ( *n* = 152, 88.4%) and intentional causes accounted for 117 cases (86%). Medical doctors were recognized as one of several toxic sources along with other medical professionals ( *n* = 26, 15.1%).

**Table 1:** Demographic characteristics of patients admitted with acute poisoning ( *n* = 172)

Variable	*N*	%
Age, mean ± standard deviation (SD)	27.3 ± 13.9	
Age category		
14–24	95	55.2
25–34	40	23.2
35–44	22	12.7
45–54	4	2.3
55–64	4	2.3
≥ 65	6	3.4
Sex		
Female	132	76.7
Male	40	23.3
Marital status		
Married	112	65.1
Single	58	33.7
Divorced	2	1.2
Job		
Housewife	91	52.9
Unemployed	32	18.6
Worker	27	15.8
Students	13	7.6
Farmer	4	3.3
Medical staff	2	1.2
Teacher	2	1.2
Soldier	1	0.6
Level of education		
Not educated	25	14.5
Primary school	62	36
middle school	65	37.8
Secondary school	12	7
Higher education	8	4.7
Smoking		
No	133	77.3
Yes	39	22.7
No. cigarettes, mean ± SD	19.1 ± 9.1	
Residence		
Urban areas	39	22.7
Rural areas	133	77.3
Type of house		
Home construction	87	50.6
Brick house within a camp	51	29.7
Tent	34	19.8
Seasons		
Autumn	43	25
Winter	44	25.6
Spring	19	11
Summer	66	38.4
Route of poisoning		
Oral	152	88.4
Dermal	5	2.9
Inhalation	13	7.6
Intravenous	2	1.2
Manner of poisoning		
Intentional	117	68
Suicide attempt		
0	153	89
1	12	7
2	4	2.3
3	1	0.6
≥ 4	2	1.2
Accidental exposure	32	18.6
Unintentional overdose	23	13.3
By mistake	18	23.7
Drug addiction	5	2.9
Source of different toxic		
Psychiatrists	5	2.9
General physician	12	7
Other medical doctor	26	15.1
Self-purchase in drugstore	27	15.7
Family, friends	33	19.2
Unknown	3	1.7

### Toxic Agents

Table 2 shows that among hospitalized acute poisoning patients drug toxins proved dominant as toxic ingestion substances ( *n* = 106, 61.6%). Organophosphorus compounds represented the second largest category of identified toxic substances ( *n* = 25, 14.5%) compared with rat killer ( *n* = 10, 5.8%) and phosphide aluminum ( *n* = 20, 11.6%), gas ( *n* = 1, 0.6%), diesel oil ( *n* = 2, 1.2%), the household chemicals Clorox and Flash ( *n* = 5, 2.9%), alcohol ( *n* = 1, 0.6%), and unidentified substances ( *n* = 3, 1.8%).

### Drug Classification

Patients who admitted with drug-related acute poisoning cases mainly took one medicine (49.1% of *n* = 52) but a smaller number took two drugs (16% of *n* = 17) and multiple drugs (≥ 3 drugs) (17.9% of *n* = 19). Eighteen patients (17% of all cases) became poisoned by drugs with unknown sources. Among the patients with drug poisonings the highest number of cases involved analgesics since 17 patients out of 107 patients (16%) experienced such drug poisonings. Eight individuals (7.5%) used nonsteroidal anti-inflammatory drugs and five patients (4.7%) used paracetamol, while a total of four patients (3.8%) used central analgesics. Psychoactive drugs led to five reported cases of poisoning, which consisted mainly of antidepressants ( *n* = 3, 2.8%) and antipsychotics ( *n* = 2, 1.9%). Among other drug classifications, as seen in Table 3 , the prevalence was substantially lesser with percentages between 0.9 and 6.6%.

### Clinical History and Presentation

The acute poisoning cases' history together with clinical manifestations and vital signs are displayed in Table 4 . The study involved 134 patients without recorded medical history alongside 11 cases with psychiatric diseases and 8 patients with cardiovascular diseases, while 7 had hypertension and 2 had diabetes alongside 1 patient with stroke and 2 with chronic kidney disease and joint disease along with 3 patients who had asthma, 3 patients had migraine, and 6 epilepsy patients and 5 pregnancies.

**Table 2:** List of toxins among patients admitted with acute poisoning ( *n* = 172)

Variable	*N*	%
Drugs	106	61.6
Organophosphorus	25	14.5
Rat killer	10	5.8
Phosphide aluminum	20	11.6
Gas	1	0.6
Diesel oil	2	1.2
Household chemicals (Clorox, Flash)	5	2.9
Alcohol	1	0.6
Unknown	3	1.8

**Table 3:** List of drugs among patients admitted with acute poisoning ( *n* = 106)

Number of drugs		
One drug	52	49.1
Two drugs	17	16
Multiple, ≥ 3 drugs	19	17.9
Unknown drugs	18	17.0
Known drug classifications		
Analgesics	17	16.0
NSAIDs	8	7.5
Paracetamol	5	4.7
Central analgesic	4	3.8
Psychoactive drugs	5	4.7
Antidepressants	3	2.8
Antipsychotics	2	1.9
Anticoagulants	7	6.6
Antibiotics	6	5.7
Antiepileptic drugs	3	2.8
Cardiovascular drugs	2	1.9
Vitamin	2	1.9
Oral antidiabetics	1	0.9
Oral contraceptive pills	1	0.9
Proton-pump inhibitors (PPIs)	1	0.9
Immune suppression	1	0.9
Muscle relaxant	1	0.9
Anticongestion	1	0.9
Antihistamines	1	0.9
Bronchodilator	1	0.9

Abbreviation: NSAIDs, nonsteroidal anti-inflammatory drugs.

**Table 4:** History, clinical manifestation, and vital signs on presentation cases of acute poisoning ( *n* = 172)

Variable	*N*	%
History		
No past medical history	134	77.9
Psychiatric disease	11	6.5
Cardiovascular diseases	8	4.6
Hypertension	7	4.1
Diabetes	2	1.2
Stroke	1	0.5
Chronic kidney disease	1	0.5
Joint disease	2	1.2
Asthma	3	1.7
Migraine	3	1.7
Epilepsy	6	3.5
Pregnant	5	2.9
Clinical manifestation		
Asymptomatic	39	22.7
Vomiting	84	48.8
Unconscious	25	14.5
Difficulty breathing	6	3.5
Dizziness	5	2.9
Headache	4	2.3
Bleeding (GI, skin)	3	1.7
Irritability	2	1.2
Salivation and lacrimation	1	0.6
Palpitation	1	0.6
Mortality	1	0.6
Vital signs		
Blood pressure (mean ± SD)	111.2 ± 23.03	
Hypotensive	14	8.1
Normal	146	84.9
Hypertensive	12	7
Pulse (mean ± SD)	97.4 ± 21.1	
Bradycardic	3	1.7
Normal	110	64
Tachycardic	59	34.3
SPO _2_ (mean ± SD)	95.1 ± 8.4	
Hypoxia	23	13.4
Normal	149	86.6

Abbreviations: GI, gastrointestinal; SD, standard deviation.

Note: The total number of diseases exceeded the total number of poisoning patients since individual patients could have multiple medical diseases simultaneously. The number of symptoms exceeded all poisoning patients because some patients experienced multiple symptoms.

In our study, family conflicts seemed to be linked to suicide attempts since 47.8% of patients reported experiencing family conflict and 11.6% experienced psychological stress. Investigation results indicated that 11% of the study group had tried to kill themselves at least once. Only 2.9% was drug abuse.

Most admissions ( *n* = 84, 48.8%) manifested through vomiting while 39 patients (22.7%) displayed no symptoms at all, and 25 patients (14.5%) suffered unconsciousness. The secondary symptoms experienced by patients consisted of dizziness, which affected 2.9% of the participants along with difficulty breathing in 3.5% as well as irritability in 1.2% and bleeding of gastrointestinal tract and skin in 1.7% and headache in 2.3%, salivation and lacrimation in 0.6%, palpitation in 0.6%, while mortality in 0.6% was reported. The blood pressure revealed a mean value of 111.2 ± 23.03 mm of mercury, while hypotension affected 14 patients (8.1%) and normotension prevailed among 146 patients (84.9%), yet hypertension developed in 12 patients (7%). The study found a mean pulse rate of 97.4 ± 21.1 with bradycardic experiencing by 3 (1.7%) patients and normal pulse by 110 (64%), while tachycardia was reported by *n* = 59 (34.3%) patients. The mean measurement of SPO _2_ amounted to 95.1 ± 8.4 and patients displayed either hypoxic conditions (13.4%) or normal SPO _2_ levels (86.6%).

### Management and Outcomes

Table 5 contains data about treatment methodologies together with hospital admission frequencies and patient residence times along with outcome evaluations. Doctors provided specific antidotes to 20 patients who comprised 11.6% of the treated population and gave symptomatic medication to 101 patients who made up 58.7% of the treated population. The health care providers used decontamination showers in 2 cases (1.1%), gut decontamination in 96 cases (55.8%), and activated charcoal in 59 patients (34.3%). The treatment protocols included oxygen therapy for 22 patients totaling 12.7% and the application of vasopressors in a single case amounting to 0.6% as well as dialysis treatment for 8 patients, which represented 4.6% of the total group. Only one patient (0.6%) received no medication. During the research period, a total of 163 patients (94.8%) received hospital care while 9 patients (5.2%) avoided admission. A total of 162 patients received care in hospital for an average duration of 33.1 hours (± 36.5), while 58 patients (33.7%) spent less than 1 day and 114 patients (66.3%) stayed for at least one day. A total of 132 patients (76.7%) received treatment that led to their full recovery and departure from the facility, while one patient (0.6%) received travel arrangements to Türkiye and 26 patients (15.1%) discharge against medical advice and 13 patients (7.6%) passed away.

**Table 5:** Management, hospitalization rate, length of hospital stays, and outcomes of patients with severe poisoning

Variable	*N*	%
Management		
Specific antidote	20	11.6
Symptomatic medication	101	58.7
Decontamination shower	2	1.1
Gut decontamination	96	55.8
Activated charcoal	59	34.3
O _2_ therapy	22	12.7
Vasopressors	1	0.6
Dialysis	8	4.6
No medication	1	0.6
Hospitalization		
Yes	163	94.8
No	9	5.2
Length of hospital stay (h), mean ± SD	33.1 ± 36.5	
< 1 day	58	33.7
≥ 1 day	114	66.3
Outcome		
Full recovery and discharge	132	76.7
Transfer to Türkiye	1	0.6
Discharge against medical advice (DAMA)	26	15.1
Death	13	7.6

Abbreviation: SD, standard deviation.

Note: The total number of patients involved in analysis exceeded group counts due to multiple treatment prescriptions assigned to individual patients.

## Discussion

Acute poisoning cases collected during July 2022 through July 2023 occurred in the northwest region of Syria. Northwest Syria faces a major medical challenge from acute poisoning that needs extensive research into solutions. A significant gap exists in published research and statistical records about acute poisoning cases due to multiple factors that include both limited medical researcher interest and data collection challenges alongside many severe medical issues deemed more pressing.

Our study findings revealed that the age range of 14 to 24 years contained the highest number of cases at 55.2%, while 76.7% of all patients were female according to research in the Arab regions and neighboring areas 5 6 7 but research in Western countries reported more male patients. 8 The study revealed summertime as the peak period for poisonings with a frequency rate of 38.4%, which aligns with similar findings from Saudi Arabia. 7

Our study revealed that toxic exposure affected mostly the rural areas in which camp residents reside (77.3%), as these communities experience obstacles in accessing suitable health care services similar to the findings of researchers from Saudi Arabia and China. 5 7

Drug toxicity accounted for 48.8% of all poisoning incidents in our study yet analgesics became the prevalent drug category that supports findings presented by five different scholarly works 5 6 7 ; however, one study showed ethanol and heroin as more common than analgesics. 8 The research is unique because it observes an excessive number of poisoning incidents involving pesticides together with rodenticides and aluminum phosphide and household chemicals. Northwestern Syria faces a major public health problem due to both unintentional and deliberate poisoning incidents, which cause this observed distribution pattern. Safe storage practices for these substances need to be reinforced because they should remain inaccessible to both children and adults alongside public education about their threats and protective steps.

Our study demonstrated that intentional poisoning emerged as the primary cause (68%) comparable to findings in the study from Turkey and China, 5 6 though the Saudi research showed a lower proportion of 35.4% of intentional cases. 7

The most prevalent symptom in our research was vomiting, 48.8%. However, data from the Saudi study indicated dizziness takes precedence as a poisoning symptom, 7 while Turkish findings displayed unconsciousness as the most prevalent symptom. 6

Acute poisoning management approached in northwest Syria differs substantially from what other researchers have documented. Eight percent of patients in northwest Syria received specific antidotes during treatment, while 38% patients in the other study received these medications. 8

Our study found that 94.8% of patients required hospital admission, which is higher than the rates reported in similar studies from other regions, such as the Saudi study, which reported an 82% hospitalization rate. 7 This likely reflects the severity of poisoning cases, limited access to early care, or possibly differences in clinical management guidelines in conflict-affected settings. Our study also recorded a 7.6% patient mortality rate, which exceeds the mortality rates in five other studies, ranging between 0.6 and 12% of cases. 5 6 7

Our research findings about suicide risk factors and triggers in patients match locally observed cases in Arab countries along with nearby regions, 9 10 even though they diverge from Western statistics which link suicide most closely to mental illness substance abuse and alcoholism. 11 12

The research delivers important information about poisoning cases in northwestern Syria while facing multiple important constraints. The collected data originated from selected hospitals across northwestern Syria, which might fail to display complete details on all poisoning cases especially in hospitals insufficient for the region. Certain instances showed incomplete accuracy for both poison dose measurements and substance types. The study's results maintain a limited scope due to its focus on patients within the age range of 14 to 24 years who are primarily female.

Follow-up research should focus on analyzing the social and psychological variables associated with poisoning incidents and assess the necessity of better psychological aid services along with targeted poison danger education programs across rural regions.

## Conclusion

Acute poisoning is a significant public health issue in northwestern Syria, predominantly affecting young, married females in rural areas. The high incidence of intentional poisoning underscores the urgent need for enhanced mental health services and public awareness initiatives.
